# Implementation of long‐acting cabotegravir and rilpivirine: primary results from the perspective of staff study participants in the Cabotegravir And Rilpivirine Implementation Study in European Locations

**DOI:** 10.1002/jia2.26243

**Published:** 2024-07-08

**Authors:** Cassidy A. Gutner, Laurent Hocqueloux, Celia Jonsson‐Oldenbüttel, Linos Vandekerckhove, Berend J. van Welzen, Laurence Slama, María Crusells‐Canales, Julián Olalla Sierra, Rebecca DeMoor, Jenny Scherzer, Mounir Ait‐Khaled, Gilda Bontempo, Martin Gill, Natasha Patel, Ronald D'Amico, Kai Hove, Bryan Baugh, Nicola Barnes, Monica Hadi, Emma L. Low, Savita Bakhshi Anand, Alison Hamilton, Harmony P. Garges, Maggie Czarnogorski

**Affiliations:** ^1^ ViiV Healthcare Durham North Carolina USA; ^2^ Centre Hospitalier Universitaire d'Orléans Orléans France; ^3^ MVZ München am Goetheplatz Munich Germany; ^4^ MUC Research GmbH Munich Germany; ^5^ University Hospital Ghent Ghent Belgium; ^6^ University Medical Center Utrecht Utrecht the Netherlands; ^7^ Department of Infectious Diseases Hôtel‐Dieu Hospital, University Paris Cité Paris France; ^8^ Paris Cité and Université Sorbonne Paris Nord, Inserm, INRAE, Center for Research in Epidemiology and Statistics (CRESS) Paris France; ^9^ Hospital Clínico Universitario Lozano Blesa Zaragoza Spain; ^10^ Internal Medicine Department Costa del Sol Hospital Marbella Spain; ^11^ GSK Collegeville Pennsylvania USA; ^12^ ViiV Healthcare GmbH Munich Germany; ^13^ ViiV Healthcare London UK; ^14^ GSK London UK; ^15^ Janssen Research & Development Titusville New Jersey USA; ^16^ Evidera London UK; ^17^ University of California Los Angeles California USA; ^18^ Center for the Study of Healthcare Innovation Implementation and Policy VA Greater Los Angeles Healthcare System Los Angeles California USA

**Keywords:** cabotegravir, healthcare professional, HIV‐1 antiretrovirals, implementation science, long‐acting injectables, rilpivirine

## Abstract

**Introduction:**

Cabotegravir plus rilpivirine (CAB + RPV) is the first complete long‐acting (LA) regimen recommended for maintaining HIV‐1 virological suppression. Cabotegravir And Rilpivirine Implementation Study in European Locations (CARISEL) is an implementation–effectiveness study examining the implementation of CAB+RPV LA administered every 2 months (Q2M) in European HIV centres. We present staff study participant (SSP) perspectives on the administration of CAB+RPV LA over 12 months.

**Methods:**

Eighteen clinics were randomized to one of two implementation support packages: standard arm (Arm‐S) or enhanced arm (Arm‐E). Arm‐S included video injection training and provider/patient toolkits. Additionally, Arm‐E included skilled wrap‐around team meetings, face‐to‐face injection training and continuous quality improvement (CQI) calls. SSPs completed surveys on the acceptability, appropriateness and feasibility of CAB+RPV LA as an intervention and its implementation into their clinics, as well as barriers and facilitators to implementation. All surveys were completed at Month (M)1 (baseline), M5 and M12; data collection was completed by February 2022. Qualitative data were obtained from semi‐structured interviews at M1, M5 and M12. The primary objective was assessed via formal statistical comparisons between study arms of the Acceptability of Implementation Measure, Implementation Appropriateness Measure and Feasibility of Implementation Measure surveys (1–5 Likert scale ranging from 1 = “completely disagree” to 5 = “completely agree”). Equivalent measures anchored to CAB+RPV LA as a therapy were also assessed.

**Results:**

Seventy SSPs completed surveys and interviews at M1, 68 at M5 and 62 at M12. Mean acceptability/appropriateness/feasibility scores were ≥3.8 (out of 5) at M12 for implementation‐ and intervention‐based measures. An analysis of covariance showed no significant differences between study arms for these outcomes. Although barriers were noted, most SSPs were not overly concerned that these would impact implementation; concern about these anticipated barriers also decreased over time. At M12, 90.3% (*n* = 56/62) of SSPs held a positive opinion about CAB+RPV LA implementation. Qualitative interviews and CQI calls highlighted three top practices that supported implementation: implementation planning; education about CAB+RPV LA clinical efficacy; and education around administering injections and managing pain/discomfort after injections.

**Conclusions:**

CARISEL demonstrated that CAB+RPV LA dosed Q2M was successfully implemented across a range of European locations, with SSPs finding implementation highly acceptable, appropriate and feasible.

**ClinicalTrials.gov number:**

NCT04399551

## 1 INTRODUCTION

Considerable progress has been made in the treatment of people living with HIV (PLWH) since zidovudine was approved in 1987 [1]. The progression of combination antiretroviral therapy (ART) has made having an HIV load below detectable limits an achievable goal for most PLWH [2]. Despite this, ART has historically required high levels of adherence to daily oral pill‐taking to maintain virological suppression and prevent the emergence of resistance [2].

Cabotegravir (CAB), an integrase strand transfer inhibitor, plus rilpivirine (RPV), a non‐nucleoside reverse transcriptase inhibitor, is the first complete long‐acting (LA) regimen recommended for the maintenance of HIV‐1 virological suppression [2, 3]. CAB + RPV LA administered every 2 months (Q2M) via intramuscular gluteal injections may address some of the challenges associated with daily oral ART, such as fear of inadvertent disclosure, anxiety related to staying adherent and the daily reminder of HIV status [4]. CAB + RPV LA was approved based on the results of Phase 3/3b trials, which demonstrated non‐inferior efficacy of CAB + RPV LA dosed every 4 weeks versus daily oral therapy, as well as of CAB + RPV LA dosed every 4 weeks versus every 8 weeks [5, 6, 7]. Both regimens were well tolerated across the Phase 3/3b clinical programme.

As this novel LA regimen represents a new treatment modality for HIV therapy, requiring regular clinical visits for the administration of the injectable treatment by healthcare providers (HCPs), logistical and resourcing adaptations, as well as additional staff training, may be required to optimize delivery. Unique challenges and nuances in the delivery of HIV care are present in every country's healthcare system; consequently, refining the strategies used to support the unique and dynamic contexts for CAB + RPV LA implementation is fundamental to successful implementation from a patient, HCP and healthcare system perspective. CAB And RPV Implementation Study in European Locations (CARISEL) is a hybrid type III [8] implementation–effectiveness study examining the implementation experiences of HCPs and PLWH over time, as well as clinical outcomes, to identify strategies to support the implementation of CAB + RPV LA dosed Q2M in various clinical settings across Europe.

Here, we present the primary implementation outcomes of the CARISEL study from the perspective of HCPs (hereby referred to as staff study participants [SSPs]) to support optimal administration and implementation of CAB + RPV LA. Secondary implementation outcomes from the patient perspective will be the subject of a separate publication. The full clinical outcomes from the CARISEL study are available and have been published separately [9].

## 2 METHODS

### 2.1 Study design and participants

CARISEL (NCT04399551) was a Phase 3b, multicentre, open‐label, hybrid type III implementation–effectiveness trial examining strategies to support the implementation of CAB + RPV LA dosed Q2M in clinical settings. The study was conducted at 18 sites across Belgium, France, Germany, the Netherlands and Spain. Clinics with no prior experience with CAB + RPV LA were preferentially selected for study participation. Both SSPs and PLWH (patient study participants) were enrolled to participate in this study—this publication focuses only on SSPs; data for patient study participants will be published separately. The study started in September 2020, during the COVID‐19 pandemic and prior to marketing authorization approval of CAB + RPV LA in Europe, and Month 12 data collection was completed by February 2022. Eligible patient study participants were ≥18 years of age, had been receiving ART for at least 6 months prior to screening and were virologically suppressed (HIV‐1 RNA <50 copies/ml). For SSPs, CARISEL was designed as a two‐arm, unblinded study with centres randomized to either standard (Arm‐S) or enhanced (Arm‐E) implementation arms (Section 2.2). The study design has been previously published [9]. From each of the 18 clinics, four SSPs of various disciplines that play a crucial role in the delivery of care to PLWH (e.g. physicians, nurses [or those administering the injections], clinic administrator/manager[s] and pharmacists) participated in the study. Implementation was carried out within the existing clinic infrastructure and no additional staff were expected to be hired in support of implementation.

The CARISEL study was conducted according to the Declaration of Helsinki principles [10]. All SSPs and patient study participants provided written informed consent. The study protocol, amendments, informed consent and other information that required pre‐approval were reviewed and approved by a national, regional or investigational centre ethics committee or institutional review board.

### 2.2 Implementation intervention

Sites randomized to Arm‐S were given the traditional provider support by a medical science lead, as well as product materials, including virtual injection training and provider and patient toolkits. Sites randomized to Arm‐E received the same support as Arm‐S with additional support, including face‐to‐face injection training, a skilled wrap‐around team (SWAT) meeting, as well as six consecutive monthly continuous quality improvement (CQI) calls. The SWAT meetings involved planning for CAB + RPV LA implementation, as well as anticipating and identifying potential barriers and solutions. Facilitated 60‐minute CQI calls were planned monthly over 6 months, from Month 2 until Month 7, with two SSPs from each clinical site in Arm‐E. The CQI process involved performance monitoring, the identification of barriers (that might ultimately affect the implementation of the treatment into clinics or the successful uptake of the treatment), the generation of potential solutions, implementation of changes to address the barriers and measurement of the success of these potential solutions. The process of addressing barriers was guided by a series of Plan, Do, Study, Act (PDSA) cycles [11]. Sites were encouraged to carry out this process in response to challenges encountered, and to work on these cycles until challenges had been resolved. Sites were also reminded of ongoing PDSAs before each call. CQI calls were facilitated by qualitative researchers (French, Spanish or English speakers) who received initial training from a CQI specialist, as well as refresher training halfway through the study. The CQI specialist also reviewed all call transcripts and provided iterative feedback to, and troubleshooting for, the qualitative researchers.

### 2.3 Clinical intervention

Patient study participants received oral CAB (30 mg) + RPV (25 mg) for 1 month to assess individual tolerability. Patient study participants then received intramuscular gluteal injections of CAB LA (600 mg) + RPV LA (900 mg) administered by an HCP at Months 1, 2 and Q2M thereafter until the study end at Month 12. Clinical and laboratory assessments were regularly performed as part of study visits to monitor safety and efficacy, including plasma HIV‐1 RNA measurements, clinical chemistries and symptom‐directed physical exams. After Month 12, patient study participants had the option to continue receiving CAB + RPV LA dosed Q2M per current clinical practice or switch to an alternative treatment regimen.

### 2.4 Outcomes and procedures

#### 2.4.1 Primary and secondary (quantitative) outcomes

The primary objective of this study was to evaluate the perceived acceptability, appropriateness and feasibility of the implementation of CAB + RPV LA from the perspective of SSPs. This was assessed through the Acceptability of Implementation Measure (AIM‐Imp), Implementation Appropriateness Measure (IAM‐Imp) and Feasibility of Implementation Measure (FIM‐Imp) surveys [12]. The primary endpoints were change from Month 1 (baseline) in AIM‐Imp, IAM‐Imp and FIM‐Imp scores at Month 12. Additionally, as secondary endpoints, the same parameters were assessed anchored to CAB + RPV LA (Acceptability of Intervention Measure [AIM‐Int], Intervention Appropriateness Measure [IAM‐Int] and Feasibility of Intervention Measure [FIM‐Int]).

Surveys were administered to SSPs at Month 1, Month 5 and Month 12. The AIM‐Imp, IAM‐Imp, FIM‐Imp, AIM‐Int, IAM‐Int and FIM‐Int are four‐item measures rated on a 1–5 Likert scale (1 = “completely disagree” to 5 = “completely agree”). Barriers and facilitators to implementation were assessed via a 31‐item survey at Month 1, Month 5 and Month 12. SSPs reported levels of concern about each potential barrier using a 5‐point rating scale (1 = “extremely concerned” to 5 = “not at all concerned”), with a higher score indicating a potential facilitator of implementation and a lower score indicating a potential barrier.

#### 2.4.2 Qualitative outcomes

Qualitative data were obtained from semi‐structured telephone interviews lasting 40–60 minutes on CAB + RPV LA implementation at Month 1, Month 5 and Month 12. Interviews followed a guide developed to support SSP‐driven discussions surrounding experience with the implementation of CAB + RPV LA. Interview questions were open‐ended and designed to elicit discussions from SSPs on broad topics. Interview topics were informed by the Exploration, Preparation, Implementation, Sustainment framework and Proctor implementation outcomes [13, 14] and included acceptability, appropriateness, feasibility and sustainability of treatment, as well as barriers to/facilitators of its implementation, and clinic processes during patient visits. Owing to the nature of this data capture, SSPs did not necessarily discuss each theme, and instead discussed the themes most relevant to their clinic. Therefore, the absence of an SSP discussing a specific topic cannot be interpreted as an indication that they agreed or disagreed with certain aspects of implementation.

Interview transcripts were prepared for thematic trend analysis using ATLAS.ti (Months 1 and 5, version 8.4; Month 12, version 9.0), a data analysis software used for qualitative research.

### 2.5 Statistical analysis

The sample size of SSPs was based on the feasibility of enrolling an adequate number of sites in each implementation arm (Arm‐E and Arm‐S), balanced with interest in testing the intervention across several types of investigative sites. Saturation was achieved (the point beyond which no new concepts were elicited), demonstrating the appropriateness of the sample size for the analysis. An analysis of covariance was performed for the statistical analysis of change in AIM‐Imp, IAM‐Imp and FIM‐Imp from Month 1 to Month 12, testing the difference between implementation arms while controlling for provider type. This analysis was conducted on the primary analysis set, which comprised all SSPs who completed both Month 1 and Month 12 surveys. All other data are reported descriptively for the full analysis set, including all SSPs who completed at least one survey at any time point (Month 1, Month 5 or Month 12).

## 3 RESULTS

### 3.1 SSP characteristics

A total of 18 sites were enrolled (Belgium, *n* = 4; France, *n* = 6; Germany, *n* = 2; the Netherlands, *n* = 2; Spain, *n* = 4), with a range of 12–50 patient study participants being treated per site. Clinics were evenly distributed across implementation arms (Arm‐E, *n* = 9 sites; Arm‐S, *n* = 9 sites). Of the 18 clinics in the study, 13 (72.2% [Arm‐E, *n* = 6/9; Arm‐S, *n* = 7/9]) had no previous CAB + RPV LA experience. In total, 70 SSPs completed the Month 1 surveys and interviews; 68 and 62 SSPs went on to complete them at Month 5 and Month 12, respectively (Table 1). The number of SSPs in each implementation arm was similar. Implementation arm and occupation for the population at each time point are summarized in Table 1. Most SSPs were nursing staff (41.1%, *n* = 29/70) or physicians (37.1%, *n* = 26/70). Overall, almost half of the SSPs (44.3%, *n* = 31/70) reported working in clinics serving between 1001 and 2000 PLWH, and none reported having clinic sizes of ≤500 PLWH.

**Table 1:** SSP characteristics

	Month 1 (*n* = 70)^a^	Month 5 (*n* = 68)	Month 12 (*n* = 62)
**Country**			
Belgium	15 (21)	15 (22)	13 (21)
France	25 (36)	23 (34)	22 (35)
Germany	8 (11)	8 (12)	8 (13)
The Netherlands	8 (11)	8 (12)	7 (11)
Spain	14 (20)	14 (21)	12 (19)
**Arm**			
Arm‐E	34 (49)	33 (49)	30 (48)
Arm‐S	36 (51)	35 (52)	32 (52)
**Occupation**			
Physician	26 (37)	25 (37)	26 (42)
Nurse	29 (41)	28 (41)	25 (40)
Admin^b^	5 (7)	5 (7)	3 (5)
Pharmacist	7 (10)	7 (10)	7 (11)
Other care provider	3 (4)^c^	3 (4)^c^	1 (2)

Abbreviations: Arm‐E, enhanced implementation arm; Arm‐S, standard implementation arm; SSP, staff study participant.

^a^ One SSP completed their survey ≥14 days after the window for data collection had closed; therefore, their data were excluded from the results. Staff dropout throughout the study was due to maternity leave, long‐term sick leave and staff leaving their team or employment.

^b^ Two of the admin staff hold a hybrid role of nurse/admin.

^c^ An error in the SSP classification was noticed during the analysis phase: two of the “Other care provider” SSPs were physicians.

### 3.2 Implementation outcomes

#### 3.2.1 Acceptability, appropriateness and feasibility of CAB + RPV LA implementation

**Figure 1:**
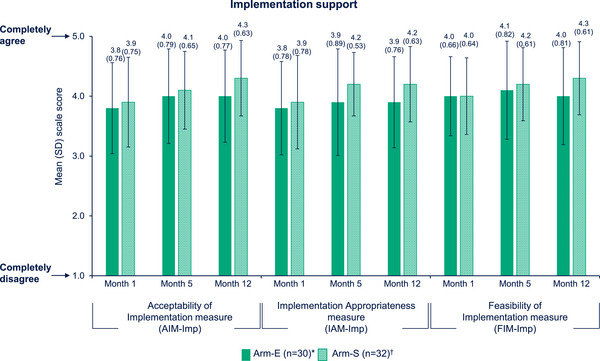
SSP perceptions of acceptability, appropriateness and feasibility of implementation of CAB + RPV LA (Survey Data). *Month 1, *n* = 34; Month 5, *n* = 33; Month 12, *n* = 30. ^†^Month 1, *n* = 36; Month 5, *n* = 35; Month 12, *n* = 32. AIM, Acceptability of Intervention; Arm‐E, enhanced implementation arm; Arm‐S, standard implementation arm; CAB, cabotegravir; FIM, Feasibility of Intervention; IAM, Intervention Appropriateness; Imp, implementation; LA, long‐acting; RPV, rilpivirine; SD, standard deviation; SSP, staff study participant.

Mean AIM‐Imp, IAM‐Imp and FIM‐Imp scores remained high for the total sample, with mean AIM‐Imp and IAM‐Imp increasing from Month 1 to Month 5 and Month 12 (Figure 1). These trends were generally consistent across both arms. In the primary analysis sets, no statistically significant differences were observed for the change from Month 1 to Month 12 between study arms, regardless of provider type (model‐adjusted mean difference between arms [Arm‐E—Arm‐S] in the change from Month 1: AIM‐Imp –0.07 [–0.48, 0.34], *p* = 0.72; IAM‐Imp –0.12 [–0.53, 0.29], *p* = 0.56; FIM‐Imp –0.31 [–0.80, 0.19], *p* = 0.22). Cronbach's alpha for the three measures overall and by arm were all ≥0.93, indicating consistent responding among SSPs.

#### 3.2.2 Implementation: Acceptability

Survey data showed that levels of SSP positivity towards implementation changed slightly over time, with the number of SSPs expressing that they were “extremely positive” or “very positive” peaking at Month 5 (80.9%, *n* = 55/68), and returning to Month 1 levels (76.8%, *n* = 53/69) by Month 12 (75.8%, *n* = 47/62) (Figure S1). Looking at the qualitative acceptability data, at Month 12, 90.3% (*n* = 56/62) of SSPs held a positive opinion about CAB + RPV LA, with 29.0% (*n* = 18/62) stating that the injectable treatment and its implementation was better than expected.

“*The truth is all of us are really very happy, all of us, starting with the patients. And we are also happy. It has been a positive experience in every aspect*.” Nurse, Arm‐E, Spain (Month 12)

#### 3.2.3 Implementation: Appropriateness

During the Month 1 interviews, SSPs discussed the potential benefits of CAB + RPV LA implementation in their clinic. Over half of the SSPs (57.1%, *n* = 40/70) discussed that the implementation of the CAB + RPV LA provided their clinic with the potential to be perceived as a leader in the treatment of PLWH. SSPs also underlined the importance of offering an innovative treatment option to their patients (Month 1: 17.1%, *n* = 12/70), as well as of offering a wider range of treatment options (Month 1: 12.9%, *n* = 9/70). Over a quarter (27.1%, *n* = 19/70) of SSPs reported experiencing pressure from their patients to be able to offer them the newest available treatments, and the same proportion (27.1%, *n* = 19/70) found it important to provide these different care options to those who have been waiting for them.

“*For the HCP, seeing them every other month creates a bond. Therefore, it is a good caregiver–patient relation*.” Nurse, Arm‐E, Belgium (Month 12)

“*For the patients, I knew that it would make their life easier but to see the relief they feel is like the cherry on top. There were also those who said that they didn't mind the pills but after the injection they realized the pressure that it put on them and that they had much more freedom now. It is good to see that*.” Nurse, Arm‐S, Belgium (Month 12)

#### 3.2.4 Implementation: Feasibility

During the Month 5 interviews, a quarter of SSPs (25.0%, *n* = 17/68) shared that implementation of the CAB + RPV LA regimen fits within the current clinic structure. Additionally, the proportion of SSPs mentioning competing clinic priorities that affected CAB + RPV LA implementation decreased from 25.7% (*n* = 18/70) during the Month 1 interviews to 22.1% (*n* = 15/68) at Month 5, and the proportion of SSPs reporting no competing priorities almost doubled from 17.1% (*n* = 12/70) to 29.4% (*n* = 20/68) over the same time period. However, resourcing issues were reported by nearly a quarter of SSPs at Month 5: 23.5% of SSPs (*n* = 16/68) reported having insufficient clinic resources to implement CAB + RPV LA post‐CARISEL and 19.1% (*n* = 13/68) indicated a need for additional staff. Nonetheless, in the Month 12 interviews, 88.7% of SSPs (*n* = 55/62) reported feeling supported in CAB + RPV LA implementation.

“*It's not hard to administer. But it wouldn't be logistically feasible to have all our patients come for injectable treatments. There are limits to our capacity*.” Physician, Arm‐S, Germany (Month 1)

#### 3.2.5 Implementation: Sustainability

During the Month 12 interviews, SSPs discussed anticipated post‐trial implementation needs, with over half mentioning staffing (61.3%, *n* = 38/62). Other anticipated post‐trial implementation needs were mentioned by fewer than half of the participants; those reported by >20% of participants are shown in Figure S2. Approximately one‐fifth of SSPs (21.0%, *n* = 13/62) indicated that they were limited in terms of scaling up implementation in their clinics. Over a third of SSPs (37.1%, *n* = 23/62) reported that some training would be needed to achieve scaling up, specifically noting that injection training (16.1%, *n* = 10/62), training for HCPs (19.4%, *n* = 12/62) and training for nurses (16.1%, *n* = 10/62) would facilitate real‐world implementation. Qualitative data suggested that some anticipated sustainability concerns may be directly related to the impact of COVID‐19 on resources.

“*We are in a particular situation with COVID. There is a major lack of nursing staff in hospitals*.” Nurse, Arm‐E, Belgium (Month 12)

### 3.3 Implementation determinants

#### 3.3.1 Barriers and facilitators

**Figure 2:**
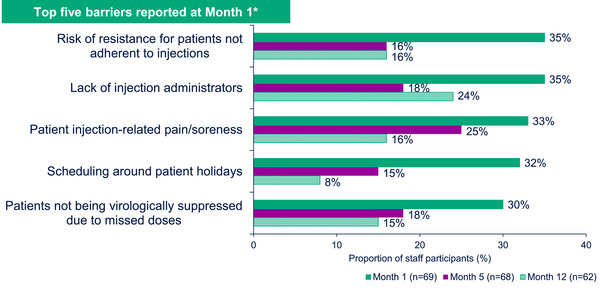
Barriers to implementation at Months 1, 5 and 12 (Survey Data). *Percentages represent the proportion of participants who were “extremely concerned” or “moderately concerned” about a potential barrier. SSP, staff study participant.

The top two barriers/concerns to successful implementation at Month 1 were: the risk of resistance for PLWH not adherent to injections and having enough injection administrators on a daily basis (both 34.8%, *n* = 24/69). Additional barriers/concerns at Month 1 included: patient injection‐related pain/soreness, scheduling around patient holidays and patients not being virologically suppressed due to missed doses. All the top five barriers reported at Month 1 markedly decreased by Month 12 (Figure 2). This trend was seen across implementation arms.

At Month 12, the highest levels of concern (“extremely concerned” or “moderately concerned”) were reported for: having enough injection administrators on a daily basis (24.2%, *n* = 15/62) and having enough resourcing for clinic flow (21.0%, *n* = 13/62). The higher levels of concern for these barriers were driven by SSPs in Germany, France and Spain. Qualitative data from interviews showed similar patterns of results. For example, in relation to staffing, feasibility barriers most often included staffing and organizational characteristics, such as workload issues and logistical aspects related to physical space in clinics for injections. Individual characteristics were largely facilitators and included experience, qualification and staff motivation. Overall, SSPs showed the least concern for confidence in the efficacy of CAB + RPV LA (82.3% [*n* = 51/62] responded that they were “not at all concerned”). While some SSPs had additional concerns around communication between pharmacy and clinic, refrigeration capacity and the oral lead‐in phase of CAB + RPV LA before starting injections, most SSPs (77.4%, *n* = 48/62) were “not concerned” about any of these.

#### 3.3.2 Implementation process

During the Month 12 interviews, SSPs discussed pre‐appointment processes, including sending appointment reminders, preparation of paperwork and arrangingn medicatio pick‐up processes with the pharmacy. In total, four administration clinic process flows were identified, which included the same five steps, ordered uniquely by clinic to optimize processes (Figure S3). The five steps were: nurse consultation, administer injections, patient observation, physician consultation and schedule next visit. Medication collection processes varied, with some SSPs (24%, *n* = 15/62) reporting that patient study participants collected their medication during clinic visits, while others (19%, *n* = 12/62) had staff collect the medication from the pharmacy prior to visits. Over half (56%, *n* = 35/62) of SSPs reported that time spent with patient study participants during visits was ≤20 minutes, and most SSPs (68%, *n* = 42/62) found time spent in the clinic for patient study participant appointments “very” to “extremely acceptable.” The majority of SSPs (76%, *n* = 47/62) spent ≤20 minutes per week ensuring appointment attendance across all patient study participants. Despite the overlap in processes, there were small variations across clinics and countries, often made to increase efficiency for SSPs and patient study participants. For example, some clinics in Germany and France gave patients prescriptions during clinic visits, allowing them to collect the medication prior to their next scheduled appointment (discussed by 19% [*n* = 12/62] of SSPs) to save time travelling between location.

**Figure 3:**
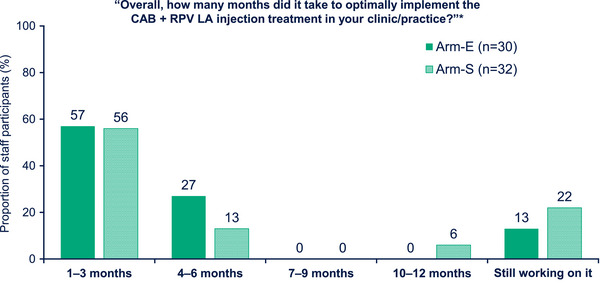
Implementation at Month 12 (Survey Data). *Data missing for 1% (Arm‐E, *n* = 1/30; Arm‐S, 1/32) of SSPs. Arm‐E, enhanced implementation arm; Arm‐S, standard implementation arm; CAB, cabotegravir; LA, long‐acting; RPV, rilpivirine; SSP, staff study participant.

At Month 12, SSPs were asked how many months it took to optimally implement CAB + RPV LA in their clinic. Over half of the SSPs (56.5%, *n* = 35/62) reported that it had taken 1–3 months to implement the new treatment; there was no notable difference between arms (Figure 3). A small percentage of SSPs reported that this process had taken between 10 and 12 months (3.2%, *n* = 2/62), and 17.7% (*n* = 11/62) reported that they were “still working on it.” Notably, a higher proportion of SSPs in Arm‐S (22%, *n* = 7/32) reported that they were still working on optimally implementing CAB + RPV LA in their clinic or practice compared with those in Arm‐E (13%, *n* = 4/30).

### 3.4 Intervention (CAB + RPV LA) outcomes

#### 3.4.1 Acceptability, appropriateness and feasibility of intervention (CAB + RPV LA)

**Figure 4:**
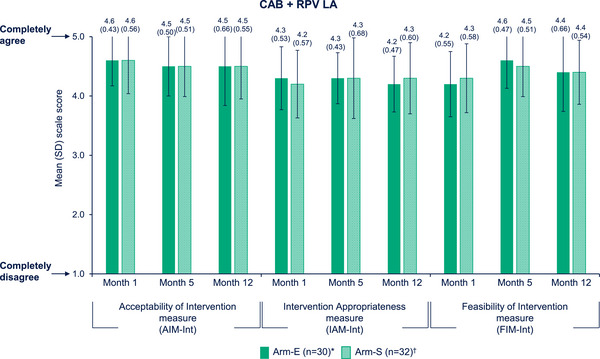
SSP perceptions of acceptability, appropriateness and feasibility of CAB + RPV LA (Survey Data). *Month 1, *n* = 34; Month 5, *n* = 33; Month 12, *n* = 30. ^†^Month 1, *n* = 36; Month 5, *n* = 35; Month 12, *n* = 32. AIM, Acceptability of Intervention; Arm‐E, enhanced implementation arm; Arm‐S, standard implementation arm; CAB, cabotegravir; FIM, Feasibility of Intervention; IAM, Intervention Appropriateness; Int, intervention; LA, long‐acting; RPV, rilpivirine; SD, standard deviation; SSP, staff study participant.

Mean (standard deviation) scores for total samples remained high across AIM‐Int, IAM‐Int and FIM‐Int through Month 12. Slight decreases from Month 1 (4.6 [0.51]) to Month 5 (4.5 [0.51]) and Month 12 (4.5 [0.60]) were observed for AIM‐Int scores. IAM‐Int scores remained stable over time (Month 1, 4.2 [0.55]; Month 5, 4.3 [0.57]; Month 12, 4.3 [0.54]). FIM‐Int scores remained high for the total sample with an increase from Month 1 (4.2 [0.57]) to Month 5 (4.5 [0.49]) and Month 12 (4.4 [0.60]). Scores were generally similar between implementation arms (Figure 4).

#### 3.4.2 Intervention (CAB + RPV LA): Acceptability

During the Month 1 interviews, SSPs discussed the potential advantages and disadvantages of CAB + RPV LA compared with daily oral therapy. For SSPs, the advantages of delivering treatment via injection outweighed the disadvantages in both the number and proportion of SSPs reporting them. SSPs mentioned several favourable attributes of CAB + RPV LA, including patient freedom and comfort (44.3%, *n* = 31/70), not being reminded of HIV status (41.4%, *n* = 29/70), the potential for increased adherence (24.3%, *n* = 17/70) and discretion for patients (17.1%, *n* = 12/70). Two of the most frequently noted potential disadvantages were the burden associated with Q2M visits (11.4%, *n* = 8/70) and patient injection pain or fear of needles (10.0%, *n* = 7/70).

At Month 12, 58.3% (*n* = 21/36) of SSPs who prescribe or recommend HIV treatments as part of their role reported no preference between CAB + RPV LA and daily oral therapy, 16.7% (*n* = 6/36) preferred prescribing/recommending daily oral therapy, and 22.2% (*n* = 8/36) preferred prescribing/recommending CAB + RPV LA. This is consistent with qualitative data expressing the need to have treatment options to individualize treatment by patient. Of note, the preferences of SSPs who do not prescribe or recommend HIV treatments as part of their role (Month 12 [40.3%, *n* = 25/62]) were not surveyed about prescribing preference.

“*I think it's [CAB + RPV LA] a nice extension of the current available regimens in HIV retroviral treatments. So, for a certain subgroup, it's certainly a very good option*.” Physician, Arm‐E, the Netherlands (Month 12)

#### 3.4.3 Intervention (CAB + RPV LA): Appropriateness

**Figure 5:**
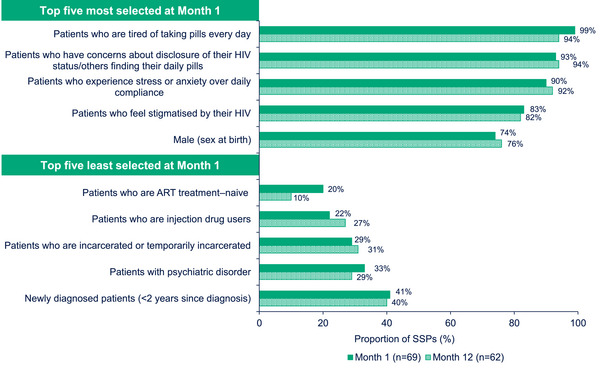
Characteristics of patients appropriate for CAB + RPV LA treatment at Months 1 and 12 (Survey Data). ART, antiretroviral therapy; CAB, cabotegravir; LA, long‐acting; RPV, rilpivirine; SSP, staff study participant.

When asked to select from a list of 24 response choices, SSPs reported that CAB + RPV LA was appropriate for PLWH with a broad range of characteristics. At Month 1, the most endorsed patient study participant characteristics were being tired of taking daily pills, having concerns about disclosure of HIV status, experiencing stress or anxiety over daily compliance and feeling stigmatized by HIV status. At Month 12, these all remained the most endorsed characteristics overall (Figure 5); 82.3% (*n* = 51/62) of SSPs endorsed being adherent to oral medication as an appropriate characteristic. At Month 12, the most frequent response (35.5%, *n* = 22/62) when SSPs were asked how many patients in their clinics they thought would switch to the injectable treatment was “between 11 and 20%.”

Qualitative data supported the idea that CAB + RPV LA was appropriate for a range of patient characteristics and that offering it as a treatment option is important.

*“I think you have to propose it to everyone because at the start you have a feeling about a patient profile that will be interested in this type of treatment, but the patient profile is in fact very diverse, and you see a lot of different types of patients you didn't expect to see who are all interested in the long‐acting medication. I think it has to be discussed with each patient and that you can be surprised about which different types of patients are interested in this.”* Physician, Arm‐E, Belgium

*“I think the type or profile of patients that might benefit from this treatment is more varied than I thought. Thus, it could probably be generalized, and the patient will be the one that will make the decision after being informed.”* Other HCP, Arm‐E, Spain

#### 3.4.4 Intervention (CAB + RPV LA): Feasibility

During interviews, SSPs discussed their experience with ViiV Healthcare toolkit materials. At Month 12, 45.2% (*n* = 28/62) of SSPs provided feedback: 17.7% (*n* = 11/62) provided positive feedback, 4.8% (*n* = 3/62) provided negative feedback and 1.6% (*n* = 1/62) reported that the toolkit was good but not all information was essential. Overall, 3.2% (*n* = 2/62) of SSPs suggested general improvements relating to live injection demonstrations and 17.7% (*n* = 11/62) reported that they did not engage with the content. When asked what was most useful about the toolkit, five (8.1%, *n* = 5/62) SSPs indicated the injection materials, with one (1.6%, *n* = 1/62) SSP each indicating the booklet and patient materials. Two (3.2%, *n* = 2/62) SSPs reported components they disliked (the online calendar and having limited information about medication).

SSPs in Arm‐E were asked about the usefulness of the SWAT meetings during their interviews. Four (13.3%, *n* = 4/30) SSPs indicated these were useful, and the primary reason cited for this was that valuable information and/or explanation about the trial was provided to them. Four (13.3%, *n* = 4/30) SSPs reported they felt SWAT meetings were not useful, with three (10.0%, *n* = 3/30) SSPs citing little value/use and one (3.0%, *n* = 1/30) SSP citing that there were too many meetings (only one SWAT meeting occurred per site in Arm‐E).

### 3.5 Additional data: CQI calls and top practice recommendations

Overall, 23 novel PDSA cycles were developed over the course of the CQI process, 12 of which were not study‐specific (52%, *n* = 12/23). The PDSA cycles that were developed were categorized into those developed in response to challenges related to logistics, systemic issues and the injection itself. Specific challenges subjected to PDSA cycles during the CQI process are shown in Figure S4.

In discussing the CQI calls, six (20%, *n* = 6/30) SSPs reported that the CQI calls helped them, and that they learnt from the sessions; however, eight (27%, *n* = 8/30) SSPs reported they did not learn anything or that they were not helpful. Some noted this was related to not feeling that they needed extra support.

*“…since we were well organized from the beginning, I did not find… it was very useful.”* Nurse, Arm‐E, France (Month 12)

**Figure 6:**
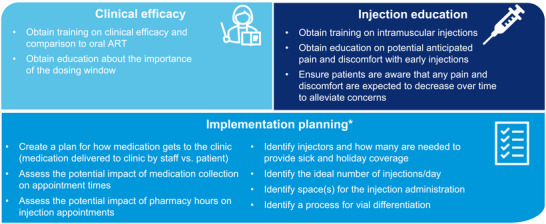
SSP top practice recommendations. *When directly asked, many felt that a planning phase has not been needed yet were able to articulate their plans. ART, antiretroviral therapy; SSP, staff study participant.

Trends emerged across sites about clinic processes during implementation from the qualitative interviews and CQI meetings. Notably, these included creating an implementation plan, educating clinic staff on clinical efficacy, as well as training staff on injection administration and the management of potential pain/discomfort post‐injection. Top practice recommendations are summarized in Figure 6. Each clinic undertook planning activities to support implementation even though not all SSPs formally identified the creation of an implementation plan.

## 4 DISCUSSION

In this Phase 3b, hybrid type III implementation–effectiveness trial carried out across diverse healthcare settings in five European countries, the implementation of CAB + RPV LA dosed Q2M was perceived as successful, regardless of implementation arm. In addition, for the clinical outcomes of the CARISEL study, CAB + RPV LA Q2M was found to be highly effective in maintaining virologic suppression, with a low rate of virologic failure [9]. The safety profile was also in line with previous findings. These results are consistent with outcomes from the CUSTOMIZE clinical trial, in which CAB + RPV LA administered once monthly was shown to be successfully implemented across a range of US healthcare settings with high rates of virologic suppression [15, 16].

The level of implementation support given (standard [Arm‐S] vs. enhanced [Arm‐E]) had no discernible impact on the measures of acceptability, appropriateness and feasibility, suggesting that CAB + RPV LA can be adequately implemented without increased levels of support. Mean acceptability, appropriateness and feasibility scores were high at Month 1 and remained high throughout the study; however, it is important to note that there were differences by arm in the time reported to reach optimal implementation, with more SSPs in Arm‐S reporting that they were still working on implementation at Month 12. This suggests that, while the level of support given may not impact perceptions of implementation, greater support may allow some sites to reach an optimal level of functioning faster than if they had received standard support. Increased support is likely not necessary for all settings but could provide an important foundation for some clinics, especially for those with higher numbers of anticipated implementation barriers.

Although barriers were noted throughout implementation, anticipated concerns decreased as SSPs gained experience administering CAB + RPV LA; however, a sizable proportion of SSPs reported having insufficient resources/staff at Month 12, as well as having concerns around limited clinic resources post‐CARISEL. This was potentially exacerbated by CARISEL coinciding with the COVID‐19 pandemic in Europe, amid regional lockdowns, when clinic resources may have been stretched or experiencing undue pressure. Taken together, these results suggest that clinical capacity is a prominent challenge for some providers of CAB + RPV LA in CARISEL. It also suggests a potentially important role of alternative sites of care to administer CAB + RPV LA when traditional settings reach capacity.

Although SSPs reported an overall positive perception, this did not translate into a greater preference for prescribing injectable treatment. Qualitative interview data did not fully explain this trend, and it could be of value to further explore barriers to prescription preference with SSPs. Despite this, it is important to remember that patient preference should be taken into account when considering CAB + RPV LA or any HIV treatment option [17].

CARISEL provided important information on process flow for the successful implementation of CAB + RPV LA. Importantly, while there are core steps for implementation, tailoring the order of steps to the clinical setting is key. Once a clinic establishes an optimal process and considers practices to support implementation (implementation planning, education about CAB + RPV LA clinical efficacy and education around administering injections and managing pain/discomfort after injection), it is easier to offer CAB + RPV LA as a treatment option to potential patients. Since CAB + RPV LA was found to be appropriate for a broad range of patient characteristics, having an efficient process in place allows it to be offered as a treatment option to a range of PLWH.

The results from CARISEL are noteworthy, as the study attempted to emulate a real‐world setting by preferentially selecting clinics with no prior experience with CAB + RPV LA for participation. The successful implementation in these, mostly LA‐naïve, clinics during the COVID‐19 pandemic is an encouraging sign as more providers adopt this new treatment modality. Additionally, the fact that 93% of all injections in CARISEL occurred within the ±7‐day dosing window, only one (0.23%, n = 1/430) patient study participant met the confirmed virological failure criterion, and 87% maintained virological suppression at Week 48 during peak waves of the COVID‐19 pandemic could also be considered a testament to the successful implementation of CAB + RPV LA [9].

### 4.1 Limitations

The small number of SSPs in some subgroups limits conclusions by country or provider type. An additional limitation is that, while the study was designed to emulate a real‐world use scenario to the extent possible, it was carried out in the context of a Phase 3b trial, removing some of the real‐world aspects. Additionally, as all centres included in CARISEL serviced ≥500 PLWH each, experience from smaller care structures is lacking.

## 5 CONCLUSIONS

Results from the CARISEL study demonstrate successful implementation of CAB + RPV LA dosed Q2M across a range of European locations, with SSPs finding implementation highly acceptable, appropriate and feasible.

## COMPETING INTERESTS

CAG, RD, JS, MA‐K, GB, RD'A, KH, HPG and MC are employees of ViiV Healthcare and may be stockholders in GSK. LH reports non‐financial support from Gilead Sciences, Merck Sharp & Dohme and ViiV Healthcare; honoraria payments and travel support for advisory board participation from Gilead Sciences, Merck Sharp & Dohme and ViiV Healthcare; and personal consulting fees from Gilead Sciences, Merck Sharp & Dohme and ViiV Healthcare, all outside the submitted work. CJ‐O reports travel supports, participation in advisory boards and activities as a speaker for Gilead Sciences, GSK, Janssen Pharmaceuticals, Merck Sharp & Dohme, Pfizer and ViiV Healthcare. LV, MC‐C and AH have no conflicts of interest to report. BJvW reports payments to institution for advisory boards from ViiV Healthcare and Gilead Sciences, and payments to institution for research funding from Gilead Sciences. LS participates in an advisory board for Gilead Sciences, Merck Sharp & Dohme and ViiV Healthcare. JOS reports participation in advisory boards and as a speaker for Gilead Sciences, Janssen Pharmaceuticals, Merck Sharp & Dohme and ViiV Healthcare, and has received research grants from Gilead Sciences and Merck Sharp & Dohme. MG is an employee of, and may be a stockholder in, GSK. NP is a contractor working for GSK. BB is an employee of, and may be a stockholder in, Janssen Pharmaceuticals. NB, MH, ELL and SBA are employees of Evidera who were paid by GSK/ViiV Healthcare to conduct the CARISEL study.

## AUTHORS’ CONTRIBUTIONS

All authors vouch for the accuracy and completeness of the data, data analyses and interpretation and fidelity to the protocol.

## FUNDING

This study was funded by ViiV Healthcare. The funder participated in the collection, analysis and interpretation of data; in the writing of the report; and in the decision to submit the paper for publication.

## Supporting information


**Figure S1**: Providers' Positivity About Implementing CAB + RPV LA at Months 1, 5 and 12 (Survey Data)
**Figure S2**: Anticipated Post‐Trial Implementation Needs Identified in Month 12 Qualitative Interviews
**Figure S3**: Sample Clinic Visit Process Flows
**Figure S4**: Challenges Discussed During the CQI Process for Which PDSAs Were Developed, by Category

## Data Availability

Data‐sharing requests will be considered by the management group upon written request to the corresponding author. De‐identified participant data or other prespecified data may be available subject to a written proposal and a signed data‐sharing agreement.
