# Supplementary material for: Implementation of long‐acting cabotegravir and rilpivirine: primary results from the perspective of staff study participants in the Cabotegravir And Rilpivirine Implementation Study in European Locations
Source: J Int AIDS Soc. 2024 Jul 8;27(7):e26243. doi: 10.1002/jia2.26243 (PMC11231444; doi:10.1002/jia2.26243)
Supplement: Supplementary file 1 — Figure S1: Providers' Positivity About Implementing CAB + RPV LA at Months 1, 5 and 12 (Survey Data) Figure S2: Anticipated Post‐Trial Implementation Needs Identified in Month 12 Qualitative Interviews Figure S3: Sample Clinic Visit Process Flows Figure S4: Challenges Discussed During the CQI Process for Which PDSAs Were Developed, by Category [file JIA2-27-e26243-s001.docx]

# Supplement

### Figure S1. Providers’ Positivity About Implementing CAB + RPV LA at Months 1, 5 and 12 (Survey Data)


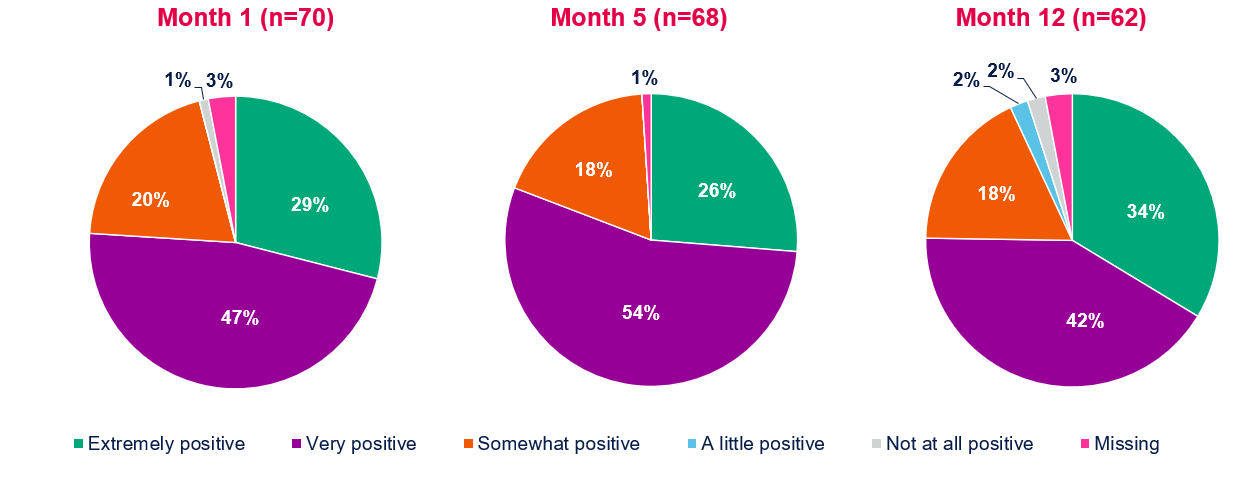


CAB, cabotegravir; LA, long-acting; RPV, rilpivirine.

### Figure S2. Anticipated Post-Trial Implementation Needs Identified in Month 12 Qualitative Interviews*


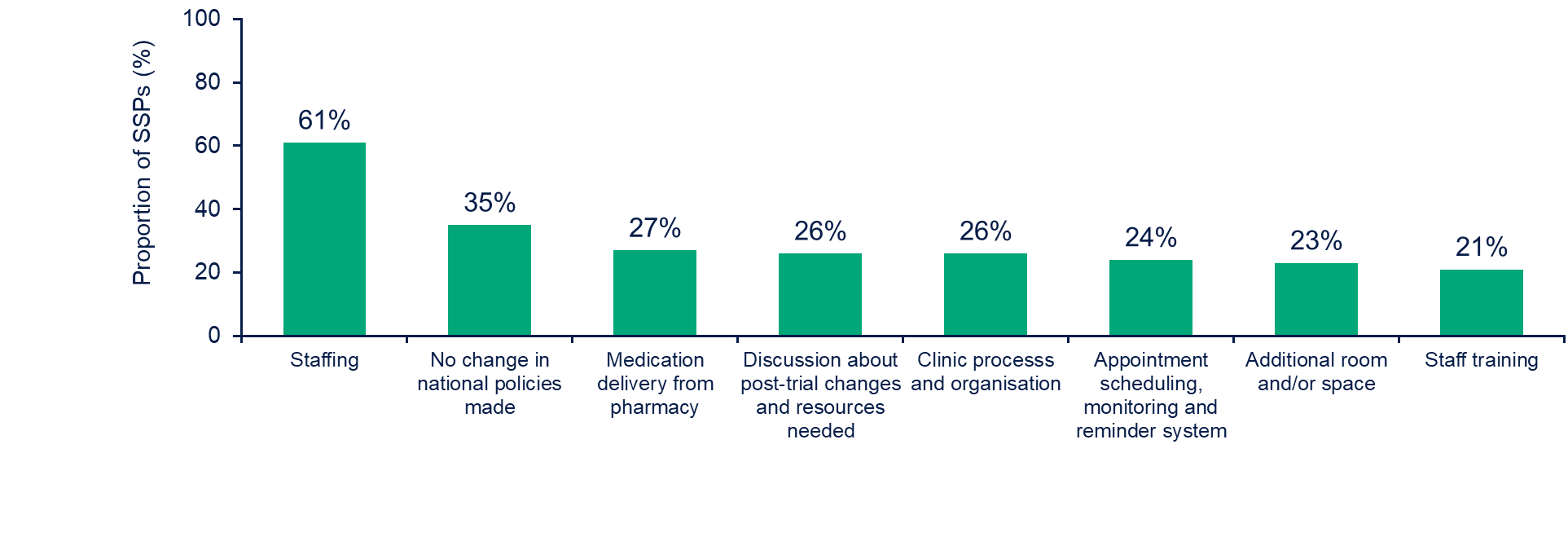
*n=62. Those reported by >20% of participants are shown.

SSP, staff study participant.

### Figure S3. Sample Clinic Visit Process Flows


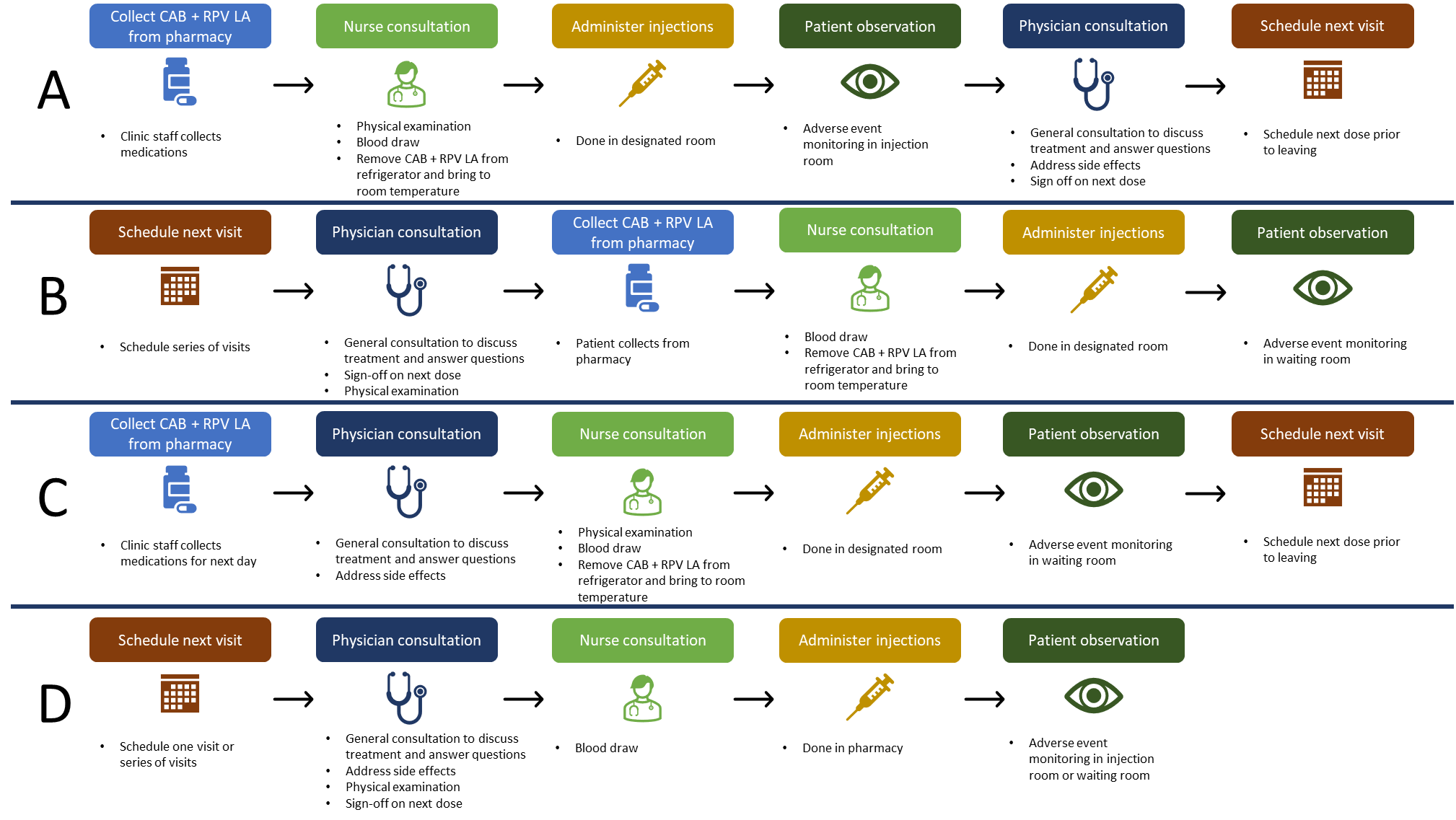


CAB, cabotegravir; LA, long-acting; RPV, rilpivirine.

### Figure S4. Challenges Discussed During the CQI Process for Which PDSAs Were Developed, by Category


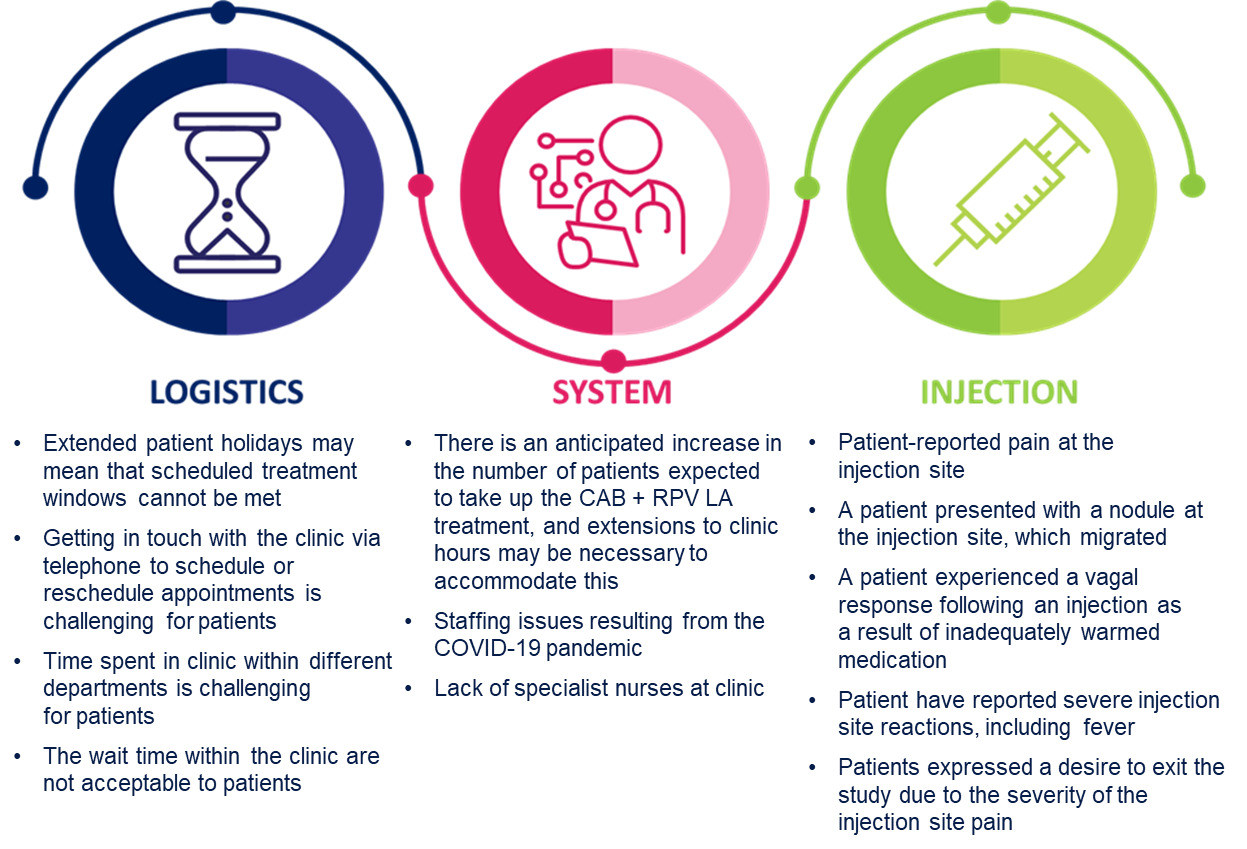
 CAB, cabotegravir; CQI, continuous quality improvement; LA, long-acting; PDSA, Plan, Do, Study, Act; RPV, rilpivirine.
